# Research-Induced Distress Among Qualitative Researchers Who Engage in Research on Child Maltreatment: A Qualitative Systematic Review of Risk and Resilience

**DOI:** 10.3390/ijerph22030329

**Published:** 2025-02-23

**Authors:** Sachet R. Valjee, Steven J. Collings, Denise Rowlett

**Affiliations:** 1 Department of Psychology, School of Applied Human Sciences, University of KwaZulu-Natal, Durban 4041, South Africa; valjees@ukzn.ac.za; 2 Independent Researcher, 3 Old main Road, Drummond, Durban Outer West 3660, South Africa; stevencollings4@gmail.com

**Keywords:** research-induced distress, sensitive topics, qualitative, child abuse, child maltreatment, researcher, risk, resilience, research ecology

## Abstract

We aimed to review and synthesize the literature on risk and resilience factors for research-induced distress (RID) among qualitative child abuse researchers, with the review guided by the Lippencott-Joanna Briggs Institute methodology for qualitative reviews. We searched Scopus, PsychINFO, MEDLINE, and ProQuest, with two researchers independently reviewing title/abstracts and full-text articles for inclusion, and with additional articles found using citation searches of identified articles and through a perusal of articles in key child abuse and qualitative research journals. We synthesized 30 unique studies, with this synthesis revealing that risk and salutary factors for RID outcomes emanate from all levels of the research ecology and, consequently, that optimal strategies for the primary and secondary prevention of RID could profitably adopt a multi-systemic perspective. Findings from this review provide child abuse researchers and members of academia with a detailed and systematic overview of potential threats and salutary influences for RID that could be used to (1) inform the development of comprehensive pre-research (and ongoing) training programmes for researchers, and (2) guide the development of secondary prevention programmes designed to mitigate RID outcomes. With respect to future research, this review suggests that the focus of research could usefully be extended in order to: (1) provide a more comprehensive perspective on the experiences of researchers living in low- to middle-income countries, and (2) ensure children’s rights to be heard, and to participate in research on matters that affect them, are more comprehensively addressed.

## 1. Introduction

There is an emerging body of research which suggests that qualitative researchers who engage in trauma-focused research face a risk for experiencing research-induced distress (RID) [1,2,3,4,5,6,7], with such distress having been found to take a number of forms including researchers’ fears for their own physical wellbeing [8,9,10,11], distressing emotional states (e.g., sadness/tearfulness, fear, and anxiety) [9,12], and symptoms of traumatic stress, including re-experiencing phenomena [4].

With respect to the definition of RID, available studies have, to a large extent, defined RID as a form of secondary traumatization that occurs in the context of intense and/or extended emotional engagement with research participants who have experienced (or are experiencing) potentially traumatic life events. Although there has been no systematic attempt to explore the relative traumatic potential of researching different types of life events, available studies suggest that RID among researchers tends to be more likely in cases where the research topic involves: child maltreatment, death or dying, violence, or natural disasters [1,4,6,11]. Although RID has been most often studied among researchers who have had direct (face-to-face) contact with research participants, it has also been noted among members of research teams who do not have direct contact with participants including: individuals who are employed to transcribe or code recorded interviews [13], researchers who examine archival material [1], and research supervisors [14].

The limited number of studies that have reported prevalence rates for RID among social researchers suggest that RID is relatively common. In a study conducted by Whitt-Woosley and colleagues [15], the prevalence rate for RID was found to be 57.7%, with qualitative researchers reporting significantly higher overall distress levels than quantitative researchers. With respect to specific types of RID, prevalence estimates for psychological distress have been found to vary from 18% to 44% [16,17], with comparative estimates for: (a) job burnout ranging from 35.5% to 54% [17,18], (b) feeling unsafe: 37.2% [16], and (c) symptoms of traumatic stress: 9.1% [16].

Available studies suggest that direct contact with trauma survivors may be sufficient to trigger distressing trauma-related countertransference reactions, with such reactions at times mirroring the traumatic experiences reported by research participants [19]. However, the mobility of trauma across time and space [20,21] has been made evident by the fact that RID has been found to be associated with: (a) prior (at times unrelated) traumatic experiences that the researcher has experienced during their lives [8,9], as well as (b) traumatic events that are not restricted to the researcher’s life-span (e.g., intergenerational trauma) [22]. Further, influences on RID have been found to emanate not only from the immediate context of research engagement but also from different spaces or places within the broader social ecology in which the researcher is embedded, including institutional and/or macrosystemic influences [2].

In sum, RID has been found to be relatively common among social researchers, with qualitative researchers and researchers who conduct research on child maltreatment facing a particularly high risk for RID [11,15]. As such, this review focuses on factors associated with risk and resilience for RID among qualitative researchers who engage in research on child maltreatment.

### 1.1. Defining and Contextualizing the Researcher

From a Conservation of Resources (COR) perspective [23,24], researchers can be viewed as individuals (with their own biographies and unique strengths and vulnerabilities) who are embedded in a network of family, interpersonal, organizational, and socio-cultural relationships; with the predictive validity of efforts designed to understand the experiences of researchers being likely to be limited if an attempt is made to separate any piece of this nested-self without reference to the greater whole [24]. In a similar vein, contemporary conceptualisations of risk and resilience suggest that the outcome of exposure to adverse life circumstances can most usefully be construed as being the product of transactions between risk and salutary influences, with such influences emanating from various socioecological systems in which individuals are embedded [25,26,27].

With respect to risk factors, RID has been found to be associated with: (a) researchers’ inexperience, lack of training and preparation, and/or past history of exposure to traumatic life events [28,29,30]; (b) events/experiences occurring during the process of engagement with participants, such as high workloads, researchers working alone, and/or threats to the researcher’s physical wellbeing [28,29,31,32]; (c) an inadequate institutional duty of care [4,33,34,35]; and/or (d) events/experiences emanating from the broader social system in which academia are embedded, including: dominant positivist research paradigms that often lead to the derision of interpretivist researchers, researching socially mediated forms of trauma such as poverty, warfare, and/or pandemic diseases, and/or national/international guidelines that do not adequately address RID among researchers [16,36,37,38,39,40]. In addition, Bracken-Roche and colleagues [36] point out that RID may be associated with a variety of researcher vulnerabilities, such as “disease or disability or other personal, societal or environmental conditions” (p. 22), that may not be directly related to research participation.

Conversely, factors/mechanisms that have been found to mitigate RID include: (a) salutary personal characteristics such as emotional intelligence, high scores on measures of resilience, and the belief that the research is important/meaningful [41,42,43]; (b) a proximal research context characterized by researchers working in pairs/teams, researchers being able to space interviews and take breaks, opportunities for discussion and informal debriefing by team members, and having/following carefully formulated safety protocols [13,44,45]; (c) an adequate institutional duty of care [4,35]; (d) having a cultural broker on the research team in cases of cross-cultural research [46]; and/or (e) the development of national and international guidelines designed to mitigate RID among researchers [36].

Available evidence suggests that concerns for a researcher’s safety are most likely to arise in situations where adequate safety protocols are not put in place (or followed) and in situations where the researcher works alone or in social contexts characterized by warfare or social unrest [31,32,37]. The likelihood of researchers experiencing distressing emotional reactions has been found to be most strongly associated with characteristics of the proximal research context (e.g., researching particularly sensitive research topics and/or the intensity of emotional engagement with participants) as well as with an inadequate culture of institutional care [33,34,40]; while traumatic stress reactions have been found to be most strongly associated with proximity encounters (i.e., researching traumatic research topics that correspond to the researcher’s own past/present traumatic experiences) [19,22,33].

Finally, there are a number of modes of contact that have characterized the relationship between researchers and participants in available RID research. Although most studies have tended to involve a researcher engaging with the stories of participants who have experienced traumatic life events [1,3,4,6,7,8,9,10,16,17,18,19,28,31,34,37,38,39,40,41,42,43] other researchers have interviewed fellow researchers to explore their experiences of RID [11,12,13,14,15,30,32,44,45,46]. However, in recent years, there have been a small number of studies emerging in which the researcher and the participant are the same person/people (i.e., autoethnographies or collaborative autoethnographies), with strategies for autoethnographic analysis including critical self-reflection, an analysis of researcher’s research journals, and in-depth discussions with fellow researchers.

### 1.2. RID Among Child Abuse Researchers

Although there are a variety of emotionally laden research topics that have been found to be associated with RID, it is generally acknowledged that research on child maltreatment poses particular challenges for researchers [47,48,49,50,51,52]; with specific challenges associated with research on child maltreatment relating to: (a) the extreme vulnerability of children [51]; a past history of child maltreatment reported by researchers [11], and anger and frustration directed at service providers who fail to adequately protect maltreated children from further/ongoing abuse [17,52].

Although much has been written about RID among child abuse researchers, there has been no systematic attempt to date to review available studies on the topic. As such, this systematic review is designed to address the question: “What are qualitative child abuse researchers’ perceptions of risk and protective factors for research-induced distress?” For the purpose of exhaustiveness, a broad definition of child abuse was employed in this review: with a child being defined as a person under the age of 19-years and with intrafamilial, extrafamilial, and structural forms of child maltreatment being considered as search terms.

## 2. Method

The protocol for this review was registered with PROSPERO prior to data collection “https://www.crd.york.ac.uk/prospero/display_record.php?ID=CRD42024593507 (accessed on 12 December 2024)”, with the review being guided by the Lippencott-Joanna Briggs Institute methodology for qualitative reviews [53].

### 2.1. Research Question

“What are qualitative child abuse researchers’ perceptions of risk and protective factors for research-induced distress?” A more detailed breakdown of the key constructs in this question was derived using the SPIDER Tool for qualitative reviews [54], that has been specifically developed and validated for use with qualitative data. A summary of this breakdown is presented in Table 1.

### 2.2. Inclusion and Exclusion Criteria

Inclusion criteria for this review were studies: (a) that employed qualitative or mixed-methods approaches (as long as qualitative data for mixed-methods studies were reported independently), (b) in which child maltreatment was either the primary focus of the research or in which child maltreatment emerged as an important research focus during data collection, (c) that reported original empirical findings relating to researchers’ experiences of their research engagement, (d) that were published at any date prior to 2024 in peer reviewed journals, and (e) that were published in English.

Exclusion criteria were studies: (a) that employed a mixed-methods approach (in which qualitative data findings were not reported independently), (b) in which child maltreatment was not mentioned as a primary or emergent research focus, (c) that did not report original empirical findings relating to researchers’ experiences of their research engagement, (d) that were published after 2023, and/or (e) that were not published in English in peer reviewed journals.

### 2.3. Information Sources

We searched MEDLINE ProQuest, PsychINFO and Scopus for studies published prior to 1 January 2024, with the search strategy being informed by four key constructs that were suggested by the research question: qualitative methods (or equivalent) AND researcher (or equivalent) AND child maltreatment (or equivalent) AND research-induced distress (or equivalent). In line with inclusion/exclusion criteria for the study, we included qualitative studies that reported original research in peer reviewed journals on any date prior to 2024, and excluded conference proceedings/abstracts, reviews of the extant literature, editorials, commentaries, and studies that were not published in English in peer reviewed journals. Specific search terms used in database searches were formulated by members of the research team, with a specialist librarian being recruited to validate the appropriateness of selected research terms. The final list of search terms used in database searches is presented in Supplementary Data S1.

### 2.4. Study Selection

Reports identified through database searches were uploaded to EndNote X9 (Clarivate Analytics, Philadelphia, PA, USA) with duplicates being removed using procedures suggested by Bramer and colleagues [55]. Title/abstract/keyword review was conducted by two researchers (S.J.C. and D.R.) who worked independently and in duplicate; with any discrepancies between researcher’s evaluations being discussed until 100% agreement was reached. In cases where discrepancies could not be resolved between researchers, a third researcher (S.R.V.) was used for blinded adjudication. Identical procedures, involving the same researchers, were employed in the full text evaluation of records for inclusion.

Two additional strategies were employed in order to identify additional records that may not have been captured in the database search. First, forward and backward citation searches were conducted in identified studies in order to identify any additional studies that were not identified in the database search. Second, the content pages of key child abuse and qualitative research journals were perused in order to identify additional studies that were published during the period 1989 to 2023 that appeared to be relevant to this review (with full-text reviews of all identified studies being conducted). Names of key journals searched are presented in Supplementary Data S2.

These procedures identified a total of 30 unique records that were included in this review [11,19,22,46,49,50,51,52,56,57,58,59,60,61,62,63,64,65,66,67,68,69,70,71,72,73,74,75,76,77], with 21 records being identified via database searches [22,46,49,52,56,57,58,59,60,62,64,65,66,67,68,69,70,73,74,75,76], seven records being identified via forward and backward citation searches [11,19,50,61,63,72,77], and two records being identified by searching key journals [51,71]. References for all identified studies are provided in the reference section and presented separately in Supplementary Data S3.

Finally, a full-text review of all 30 identified reports confirmed that all reports addressed child maltreatment (or equivalent) and RID among qualitative researchers, However, none of the nine studies that were identified through citation searches or journal searches included key search terms—child (or equivalent) or child maltreatment (or equivalent)—in the title, abstract, or keywords, with this probably being the reason why they were not initially identified during the database search.

**Figure 1:**
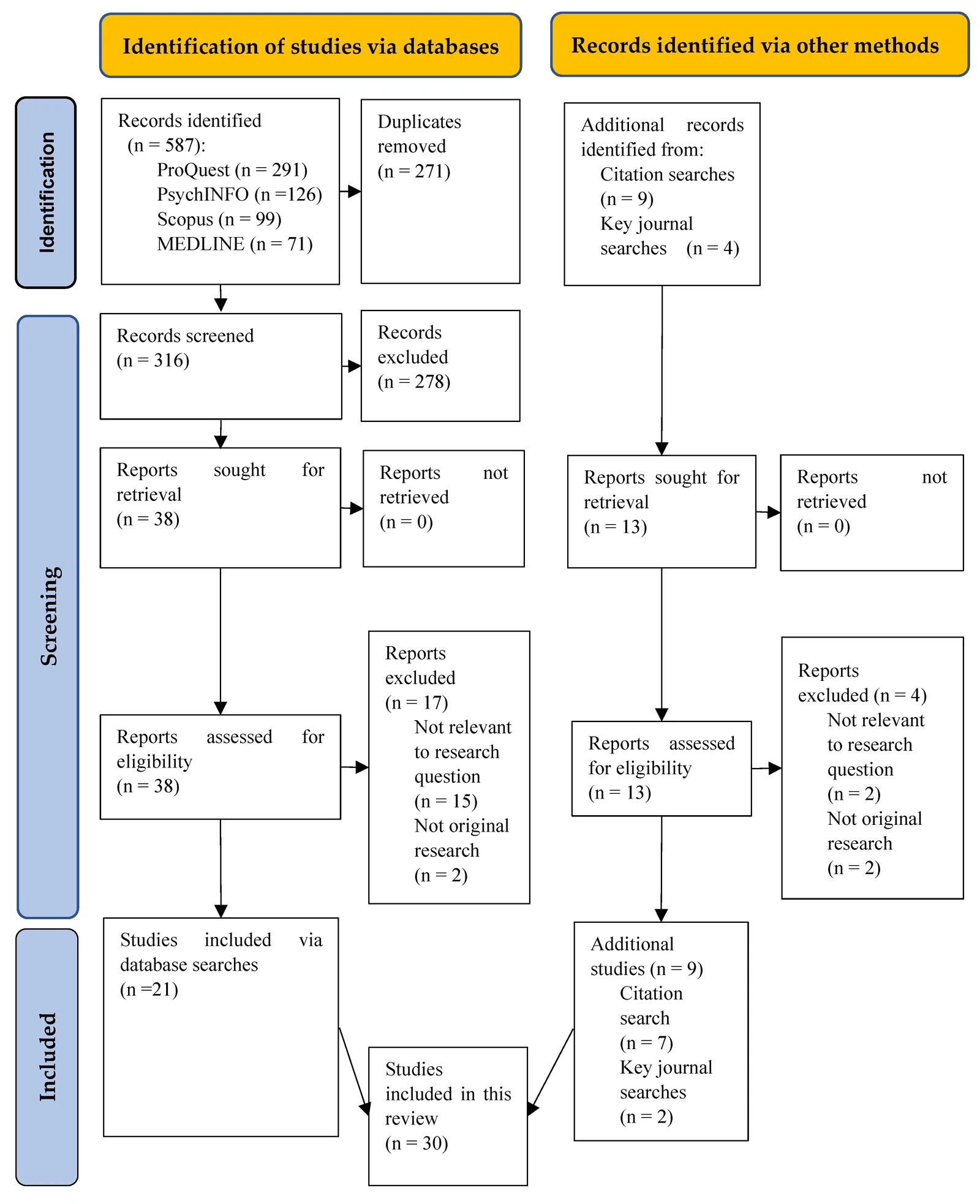
PRISMA 2020 flow diagram.

The PRISMA flow diagram for this selection procedure (Figure 1), was guided by the framework proposed by Page and colleagues [78]. This updated framework provides systematic procedures for decision-making regarding the inclusion of identified study records, with the PRISMA template for searches that involve both database and additional records (obtained via other methods) being employed in this study.

### 2.5. Data Extraction

The following data were extracted from studies that were identified for inclusion in this review: (1) Researcher characteristics (number of researchers, discipline, geographical location, continent, country, country income level, research experience); (2) Study design (types of child maltreatment studied, participants, sampling strategy, research design, and data reduction strategies; and (3) Researchers’ perceptions of risk and salutary influences on RID outcomes. An initial data extraction sheet was developed by one researcher (S.J.C), with this sheet subsequently being adapted and augmented based on feedback provided by researchers during regular team meetings.

### 2.6. Data Synthesis

Data synthesis was conducted using a three-stage thematic synthesis approach for synthesizing qualitative data [53,79]. Stage 1 of this approach involved a line-by-line coding of all reviewed studies in order to identify phrases, sentences, or paragraphs relating to the wellbeing of researchers; with words/phrases/sentences conveying the same meaning (e.g., crying or tearfulness) being combined into one code, and words/phrases/sentences conveying distinctly different meanings (e.g., feeling tearful versus feeling angry) being coded separately. Coding was conducted by two researchers (S.J.C. and D.R.) who worked independently, with research questions being put aside during coding in order to minimize an imposition of an a priori framework on the coding process. Finally, coders met to compare identified codes, with coding discrepancies being resolved through discussions between coders until a consensus was reached. Finally, a team discussion, involving all researchers, was arranged to confirm that identified codes were exhaustive and that there was consistency in coder interpretation.

The second stage of the synthesis involved the development of descriptive themes, which entailed examining similarities and differences between initial codes in order to identify new (higher level) themes that captured the meaning of groups of codes. Each researcher in the team did this independently, prior to group discussions, designed to achieve consensus, and to ensure that all initial codes were adequately addressed by identified descriptive themes.

Finally, the third stage of the synthesis was designed to generate analytic themes. This superordinate level of synthesis was achieved by using the descriptive themes that emerged during Stage 2 of the synthesis to derive analytic themes that went beyond the content of reviewed studies to address the review question more directly (that had temporarily been put to one side during earlier stages of synthesis). Identified analytic themes (i.e., risk versus resilience and an eco-systemic framework) were derived from identified descriptive themes and informed by contemporary conceptualizations of stress, coping, and resilience [2,23,24,25,26,27], with these analytic themes being designed to ensure that the research more directly addressed the review question.

This process of data synthesis was iterative in nature, with adaptations, modifications, and additions being made following team discussions during the course of the data synthesis.

### 2.7. Quality Assessment

The quality of studies was assessed using the Joanna Briggs Institute Qualitative Assessment and Review tool (JBI-QARI) [80], that contains 10-items which focus on the rigour of research design and the quality of reporting. For this review, JBI-QARI evaluations were conducted independently by two researchers (S.J.C. and D.R.) with blinded adjudication by a third researcher (S.R.V.) being employed in cases where consensus could not be reached. Studies were generally rated as high quality, with 25 studies meeting 8–10 quality criteria [11,22,46,49,50,51,57,58,59,60,61,62,64,65,66,67,69,70,71,72,73,74,75,76,77] and 5 studies meeting 6–7 criteria [19,52,56,63,68]. Quality ratings for each study are presented in Supplementary Data S4. No studies were excluded based on quality assessment.

## 3. Results

### 3.1. Study Descriptions

A summary of study descriptions is provided in Table 2.

**Figure 2:**
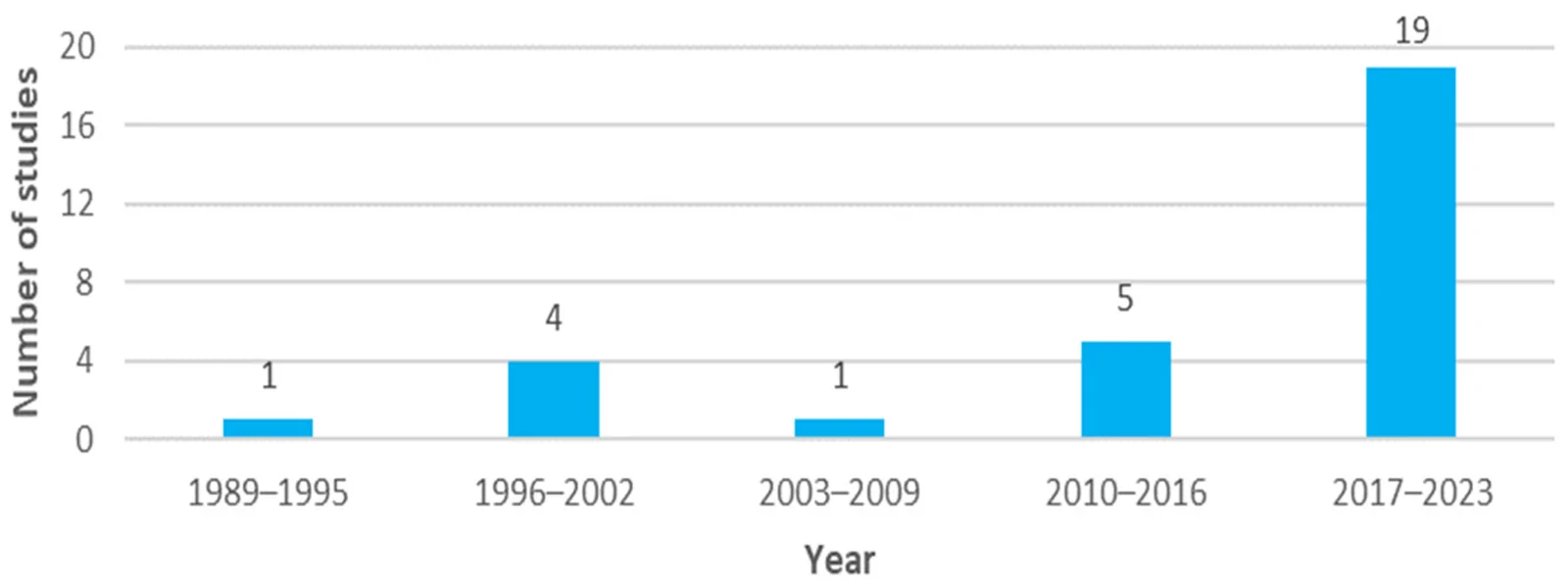
Number of studies by year of publication.

The 30 studies that were included in this review reported on the perceptions of 156 qualitative researchers. Although the review included studies published over a 35-year period (1989 to 2023), a breakdown of studies by publication year (Figure 2) indicates that the majority of studies (63.3%) had been published in the past seven years, providing a largely contemporary perspective on RID.

Researchers’ home discipline was predominantly the social sciences (53.3%) or health sciences (36.7%), with the majority of researchers self-identifying as female (71.8%). According to World Bank ratings [81], three studies reported on the perceptions of researchers residing in middle-income African countries (South Africa and Tanzania) [22,46,50], with all other studies reporting on the perceptions of researchers residing in high-income countries, and with no studies reporting on the perceptions of researchers’ living in low-income countries. Identified studies were conducted among researchers in four continents—North America (42.9%), Oceania (28.2%), Europe (24,4%), and Africa (4.5%)—with two studies [46,73] involving research teams that included researchers from more than one continent. With respect to the researchers’ country of residence, approximately nine out of 10 studies (92.9%) were conducted in one of four countries: the United States (27.6%), Australia (27.6%), the United Kingdom (22.4%), or Canada (15.4%).

In 13 studies (43.3%) definitions of child maltreatment were restricted to one or more of the four classical types of child maltreatment (physical, sexual, emotional, and/or neglect) [11,21,49,56,57,60,63,67,70,71,72,75,76], with additional forms of child maltreatment being considered in a number of studies, including: filicide [50,51,61,73], witnessing domestic violence [58,68], child labour [46], household poverty [22,46], intergenerational trauma [22,66], unaccompanied child migrants [74], or the commercial sexual exploitation of children [68]. In the remaining six studies child maltreatment was (or emerged as) a research focus, without any specific type/s of child maltreatment being specified in the text [52,58,62,64,65,77]. With respect to data sources, information relating to child maltreatment was obtained from face-to-face interviews with: children [46,52,58,64,65,67,68,74], caretakers of abused children [51,56], adult survivors of child maltreatment [11,22,46,49,50,57,58,59,60,63,64,66,69,71,76], and/or neighbours of maltreated children [61]. In seven studies, no face-to-face interviews were conducted, with researchers obtaining data relating to child maltreatment exclusively through file or archive reviews [21,62,70,71,72,75,77].

With respect to research methodology, all studies employed convenience sampling and cross-sectional designs, with data reduction strategies taking a number of forms, including: thematic analysis [11,21,52,56,63,65,68,73,74,77], a process of critical self-reflection [11,21,52,56,63,65,68,73,74,77], and autoethnographic analysis [22,61,66,67,69,70,75].

### 3.2. Data Synthesis

The hierarchical structure that emerged from the data synthesis is presented in Table 3. Line-by-line perusal of identified reports identified 115 codes, with these codes being more or less equally divided between risk factors for RID (*n* = 58, 50.4%) and salutary influences on RID outcomes (*n* = 57, 49.6%). From Table 3, it is evident that approximately two out of three codes related to events or experiences that occurred in the proximal research domain (*n* = 78, 68.4%) that encompasses the nature of the research topic, informal support from team members, and potential threats to researchers’ wellbeing emanating from research engagement. The three most frequently mentioned risk factors for RID were: child sexual abuse as a research focus (*n* = 20 reports, 66.7%), researchers’ past experience of exposure to traumatic life events (*n* = 18 reports, 60.0%), and researchers working alone (*n* = 15 reports, 50%); with the three most frequently mentioned salutary influences being regular team member discussions (19 reports, 63.3%), researchers having ≥5-years research experience (17 records, 56.7%), and informal peer debriefing sessions (14 reports, 46.7%).

A total of 39 descriptive themes emerged from the data synthesis, with these descriptive themes relating to risk factors for RID (21 themes, 53.8%) or salutary influences on RID outcomes (18 themes, 46.2%). Finally, consistent with contemporary conceptualisations of traumatic distress [23,24,25,26,27] and contemporary conceptualisations of RID [2], two themes (risk versus resilience, and the influence of different ecosystemic level on RID outcomes) were considered as analytic themes.

### 3.3. Child Abuse Researchers’ Perceptions of Risk Factors for RID

This section explores researchers’ perceptions of risk factors for RID, with a detailed description of researchers’ experiences—including rich and verbatim accounts of researchers’ perceptions identified during line-by-line coding—presented in the text, and with a more extensive list of researcher’s verbatim comments being provided in Supplementary Data S5.

With respect to levels of the research ecology that emerged in the data synthesis, child abuse researchers’ perceptions of triggers for RID can be considered in relation to triggers relating to: (a) the researcher domain, (b) the proximal research domain, (c) the intermediate/institutional domain, and/or (d) the broader socio-cultural context in which research institutions are embedded.

#### 3.3.1. The Researcher Domain

For many researchers, a personal lack of research experience (<5 years research experience) was perceived to be associated with a greater risk for experiencing RID [28,46,49,50,56,57,60,66,69,70], with a lack of secure tenure being mentioned as a risk factor for RID by researchers who were employed on a fixed-term or casual basis [74,75].

A lack of preparedness—limited prior research experience and/or inadequate pre-research training—was perceived to pose a risk for RID [11,22,28,46,49,50,51,52,56,57,58,59,60,61,65,66,67,69,70,71,72,74,75], with such outcomes tending to be more likely in cases where the researcher was: an early career researcher (<5-years research experience) [28,49,50,56,59,60,66,69,70] and/or where the researcher was employed on a fixed-term/casual basis (73,74). However, a number of researchers expressed the view that experienced researchers would also benefit from more adequate training and preparation [52,71,73]. For example:

*“…in this project, the young co-researchers appeared to be at a loss when faced with traumatic stories during interviews”*

[46]

*“The RAs (research assistants) were untrained in data analysis, being post-graduate students who had not before undertaken any research with sensitive subjects or had experience coding interview data. Nonetheless, they were asked to code these interviews within a short period of time in order to ensure we remained within our deadlines”*

[75]

*“Only at the end of data collection did I realize there is literature where researchers discuss fieldwork that can be psychologically and emotionally wrenching for investigators regardless of how experienced they are in conducting research”*

[71]

#### 3.3.2. The Proximal Research Domain

Consistent with Coddington and colleagues’ perspective on traumatic contagion [20,21], researchers’ perceptions of key triggers for RID encompassed not only forms of traumatic countertransference that occurred as a result of listening to or reading about survivors’ experiences but also reflected “*connections to other and often unrelated traumas in other times and spaces*” (p. 68) [20].

With regard to traumatic countertransference, all 30 reports provided evidence that listening to or reading detailed accounts of children’s abuse experiences had the potential for triggering RID. Further, and consistent with the views of Figley [82], two reports [19,66] highlighted the fact that countertransference reactions of researchers often mirrored traumatic symptoms reported by research participants. In addition to evoking emotional distress, the process of engaging with the experiences of abused children also posed a threat to some researchers’ sense of safety in their homes and/or raised concerns about the safety of the researchers’ children [19]. For example:

*“After reading accounts of child maltreatment, staff voiced feelings of anger, sadness, helplessness, and frustration”*

[52]

*“Many of the reactions reported by the researchers—sleeping disorders, emotional changes, somatizing, increased cautiousness, and the need for social support—closely parallel reactions experienced by…victims”*

[19]

*“In reviewing the cases, I became more and more frightened, as I continued to find young mothers who were attacked in their homes. The threat of harm to their children was often the submitting factor. As a mother, I felt anger and sadness that this happened to these women, and fear in the realization that it could happen to me”*

[19]

The view that trauma is mobile across place and time [20,21] was reflected in the fact that many researchers reported intrusion phenomena in terms of which traumatic memories—either explicit (conscious) or implicit (subconscious)—relating to events that occurred in other places and times were activated or re-activated as a result of engaging with abused children’s stories. Explicit memories of past abuse experiences were reported by two researchers:

*“Anna told me the first time a client brought her story of her childhood abuse, Anna ‘froze’. She was stunned at the similarity in their stories: her client was molested by a man, also in a confined space in a public area”*

[66]

*“Rebecca’s sense of relating to Jasmine, particularly with regard to the lasting emotional and existential impacts of her abuse experiences, made this interview data particularly traumatic to engage with and tested her emotion regulation skills”*

[49]

One researcher reported that research engagement evoked explicit memories relating to events that occurred prior to the researcher’s birth (i.e., gross violations of human rights experienced by a parent in the past):

*“This is really personal, but my mother is a concentration camp survivor and as a child, she had to flee and was caught. And so, for me, I think that this issue of what’s going on right now is excruciating for me in a really deeply personal way”*

[74]

In addition, there was one researcher who reported that implicit memories of abuse only emerged as explicit memories in the context of research engagement:

*“…shortly after I began collecting data, I started to have panic attacks and found myself unable to continue recruiting subjects. Subsequently, I developed flashbacks and other symptoms that I eventually recognized as memories of being sexually abused as a child”*

[60]

#### 3.3.3. The Intermediate/Institutional Research Domain

A number of researchers identified an inadequate duty of care on the part of institutions (ethics review boards, team leaders, supervisors, academic colleagues) as a factor that either triggered or compounded RID outcomes [3,4,5,6,7,8,9,10,11,12,13,14,15,16,17,18,19,20,21,22,23,24,25,26,27,28,29,30,31,32,33,34,35,36,37,38,39,40,41,42,43,44,45,46,47,48,49,50,51,52,53,54,55,56,57,58,59,60,61,67,70,72,75,77]. For example:

*“I felt misunderstood and alone in my work. The lack of support during the research phase of my work exacerbated my feelings of agitation, anxiety and fear…There was no peer or supervisor to talk with. I was completely alone”*

[67]

*“Although senior leaders within the research project did offer to debrief with me, I felt uncomfortable in discussing my experiences of trauma and the impact of the work with them. In large part, this was due to their lack of knowledge in being able to appropriately and efficiently support me”*

[75]

*“Safety of participants was the Ethics Committees’ primary consideration. However, while the researchers’ physical safety and supervision requirements were addressed, ‘emotional’ safety considerations were not directly dealt with”*

[58]

In addition, many researchers referred to an absence of adequate pre-research training as a trigger for RID during research engagement [11,22,51,52,57,58,61,65,67,71,72,74,75]. For example:

*“Many participants described their lack of training as being blindsided—where the expectations of engaging with data did not always align with their actual experiences”*

[11]

*“Strategies should be in place to minimize the risk of psychological impact, and quality training and supervision supports researchers to feel adequately equipped to carry out research effectively and sympathetically”*

[52]

#### 3.3.4. The Distal Research Domain

An important trigger for RID related to the fact that qualitative/interpretative methods fail to comply with the dictates of dominant positivist perspectives on social science research that emphasize objectivity and emotional detachment. As such, a number of researchers engaged in a process of what Hochschild [83] refers to as ‘surface acting’, in terms of which researchers’ regulated the public expression of their emotions in order to avoid disapproval from the academe and/or significant others [70,72,74,75]. For example:

*“Academics are expected to have control of their emotions and to be professional and rational…I was reluctant to talk to someone about what I had been reading…I was worried I would be criticized, either in a judgmental way for selecting such a topic to study, or for being a complainer or a ‘weak’ person if I shared my concerns of how the content troubled me”*

[70]

*“The power dynamics at work mean that researchers who are reliant on variable and uncertain work may be reticent to report their experiences of trauma to their manager, for fear of being taken off the research project and losing income”*

[75]

Researchers’ comments regarding socio-cultural triggers for RID largely focused on the researchers’ role as a cultural outsider, with a number of researchers suggesting that being a cultural outsider is likely to impact negatively on the researchers’ wellbeing and/or on the research process [46,64,65]. For example:

*“A further member had researched sexual and intimate partner violence in diverse cultural settings. For the research assistants involved in the research conversations, transcription and analytical phases elicited emotional responses to the work”*

[64]

*“A decline in interpreter performance…can be heightened in situations where there are cultural taboos preventing transfer of information (e.g., taboos around sexual detail being discussed with the opposite gender)”*

[65]

However, one researcher maintained that being a cultural insider may be associated with an increased risk of exposure to RID during the process of research engagement:

*“A subtheme that emerged was how participants were affected when they closely identified with the participant due to sharing similar characteristics, cultures, or experiences. For example, Participant 4 stated “I think that…we have to be more careful when our participants look like us”…It’s so much easier to personalize when the person looks like me or sounds like me”*

[11]

### 3.4. Researchers’ Perspectives on Salutary Mechanisms Mitigating RID Outcomes

This section explores researchers’ perceptions of salutary factors mitigating RID outcomes, with a detailed description of researchers’ experiences—including rich and verbatim accounts of researcher’s perceptions identified during line-by-line coding—presented in the text, and with a more extensive list of researcher’s verbatim comments provided in Supplementary Data S6. As was the case for risk factors, salutary mechanisms that were perceived to mitigate RID outcomes were identified at all levels of the researchers’ research ecology.

#### 3.4.1. The Researcher Domain

Discussions of salutary researcher characteristics largely focused on active coping styles (e.g., engaging in self-care activities or employing self-help coping strategies) as a protective factor [11,22,49,50,51,56,58,59,61,62,63,64,66,67,68,69,72,73,74,75,76,77]. Additionally, some researchers reported resilient traits and characteristics (general resilience, spiritual beliefs, research experience) that served to mitigate RID outcomes [11,22,49,58,69,70,71,73]. For example:

*“I have learnt to take my emotional safety seriously. I access clinical supervision; I draw on the support of my colleagues; and also, my family and friends where needed… That anchors me”*

[73]

*“Throughout this autoethnographical piece and my life, resilience has been a recurring theme displayed by my mother, myself and my family”*

[69]

#### 3.4.2. The Proximal Research Domain

There were a number of researchers who felt that the process of research engagement led to the development of personal resilience [59,64,69,70,71,73]. In two studies, an emerging sense of researcher resilience was directly inspired by the resilience of research participants [59,70]. For example:

*“I can now appreciate and be thankful for the resilience which I have developed through the PhD, and the ability it provides me to empathize with others who are researching emotionally difficult and sensitive subject areas”*

[70]

*“Carina’s interview was…another encounter that proved meaningful for her and for me as well…To me, Carina’s way of living her life was a metaphor for resilience, broadly defined as “the capacity to rebound from adversity strengthened and more”*

[59]

#### 3.4.3. The Intermediate/Institutional Research Domain

Researchers perceptions of factors mitigating RID related largely to ongoing training received during the process of research engagement [45,51,58,59,61,62,63,68,70,71,72,76], adequate academic and clinical supervision [11,22,49,51,58,59,62,68,71,76,77], and helpful ethical guidelines provided by members of the academe [49,57]:

*“What worked particularly well for this group was formally provided and appropriate supervision, support and training”*

[71]

*“With the assistance of the University Ethics Committees, we carefully planned responses to participant distress”*

[58]

#### 3.4.4. The Distal Research Domain

Efforts to counter the effects of stoic professionalism associated with surface acting involved researchers’ efforts to share their emotional burden in safe places (mainly in discussions with colleagues and team members) [70,72]. For example:

*“I serendipitously ran into a former colleague, who offered to be a listening ear if ever I needed it. I took up the offer and we met for coffee one afternoon several weeks later. I was permitted to share of my experience, sorrows, frustrations (at institutions) and anxieties. There was no judgement, only compassion and understanding. This was the starting point for me to process and reconcile all that I had learned, read, felt and heard”*

[70]

With respect to addressing triggers for RID in cross-cultural research, one study employed a number of strategies, including obtaining advice and guidance from local children’s rights experts and ensuring that cultural brokers formed part of the research team:

*“I refined the research project based on advice from Tanzanian children’s rights experts…(and extended)…the ‘right’ to participate in research projects to non-academics by insisting that affected communities and individuals (including maltreated children) be involved in all possible stages of the research process and associated outcomes”*

[47]

## 4. Discussion

The aim of this research was to explore child abuse researchers’ perceptions of risk and salutary factors that impact on their wellbeing. Consistent with findings from previous studies [1,2,4], the present findings suggest that influences on RID can usefully be considered in relation to a number of different layers of influence with these layers encompassing not only the researcher but also multiple levels of the research ecology in which the researcher is embedded, with the responsibility for addressing RID and consequently also needing to be considered at all levels of the researcher’s social ecology [2].

The data synthesis produced a contextualized framework that was exhaustive (i.e., all identified triggers for RID were accommodated in the framework), with the heuristic value of the framework being suggested by the fact that factors relevant to RID outcomes were identified in each of the content domains defined by the two analytic themes (levels of the research ecology and risk versus resilience mechanisms) included in the data synthesis.

It is notable that some identified salutary factors or mechanisms were characterized by multifinality, with certain strategies (e.g., seeking professional counselling) being perceived by researchers as mitigating RID outcomes in relation to risk factors occurring at a number of different levels of the research ecology. This finding is not particularly surprising as levels of the research ecology are not regarded as being distinct, with the outcome of a particular salutary influence being regarded as being shaped by the interactions and coactions of many systems working in concert [25].

With respect to the dynamics of RID, review findings suggest that researcher wellbeing is not simply a function of influences emanating from different levels of the researchers’ social ecology but may also involve synergies between multisystem risk and protective factors working in concert [27]. Thus, for example, accounts of intrusion (or re-experiencing) phenomena reported by researchers in this review would clearly appear to involve synergies between factors emanating from the both the researcher domain (a past history of traumatic exposure) and the proximal research domain (researching topics that are particularly salient in terms of the researcher’s biography). As Masten and colleagues [27] point out, such synergies suggest the need for a multisystemic perspective that not only acknowledges the influence of multisystemic influences on individual’s wellbeing but also addresses synergistic transactions between different eco-systemic levels and/or different risk and protective factors. Although only a few of the studies in this review considered risk and protective factors emanating from more distal systemic influences (cultural and chronosystemic influences), available studies suggest that such distal influences have the potential to exert significant synergistic influences on individuals’ wellbeing [20,21,84,85,86], and therefore need to be more comprehensively addressed in future studies of RID.

With respect to the implications of this review for researchers, the review provides child abuse researchers with a broad range of risk and salutary factors that can be used by researchers to prepare them for potential RID and to provide them with strategies that can be used to mitigate RID. Similarly, the findings from this review could usefully be considered by research institutions in their drafting of institutional protocols designed to protect the physical and psychological wellbeing of child abuse researchers.

Finally, it needs to be considered whether risk and protective factors for RID among child abuse researchers are similar to, or different from, factors that have been found to be associated with RID outcomes in the general literature on RID. The answer to this question is quite simply that in the vast majority of cases, risk and protective influences identified in this review are similar to influences identified in previous studies. However, some influences appear to be largely unique to child abuse researchers (e.g., researchers increased concerns relating to their child being maltreated or re-experiencing phenomena related specifically to the researchers’ past history of child abuse). Conversely, it is of course possible that risk and salutary influences identified in the general literature on RID may include additional influences that were not identified in this review.

### Strengths and Limitations

We believe that this review has a number of strengths. To the best of our knowledge, this review represents the first attempt to explore factors associated with RID among qualitative child abuse researchers, with: (a) the research protocol for the review being registered with PROSPERO prior to the commencement of the review (CRD42024593507), (b) searches being conducted using four databases (Scopus, MEDLINE, PsychINFO, ProQuest), augmented by full-text citation searches of all identified reports and additional searches in key journals, (c) study selection procedures being informed by the updated PRISMA 2020 statement [77], and (d) the review being guided by the Lippencott-Joanna Briggs Institute methodology for systematic qualitative reviews [53].

A further strength of this review is that it provides social researchers with a broadened conceptualization of RID that encompasses a view of the researcher as being embedded in multiple levels of the research ecology, as well as an extended perspective on transactional and synergistic associations between risk and salutary factors at different systems and levels. These findings would appear to have clear implications for future research on RID in relation to the ways in which such research is conceptualized and designed, as well as the ways in which research findings are interpreted.

However, the inclusion criterion that restricted all searches to records published in English may have excluded some potentially relevant studies. Further, the fact that 95.5% of reviewed studies reported on the perceptions of researchers living in high-income countries suggests that study findings may not be generalizable to researchers living in low- or middle-income countries. Finally, given that all authors of this paper are experienced child abuse researchers, it is possible that our own perceptions of researching child abuse may have unintentionally influenced study findings. Although concerted efforts were made by all authors to avoid such bias, we cannot be certain that we were totally successful in these efforts.

## Figures and Tables

**Table 1:** A breakdown of key constructs from the research question using the SPIDER Tool.

Construct	Search Terms
S—Sample	(“researcher”) OR (“interview *”) OR (“transcri *”) OR (“coding”) OR (“coder *”) OR (“interpret *”) OR (“translat *”) OR (“supervisor”) OR (“research team”)
PI—Phenomenon of interest	(“research-induced distress”) OR (“vicarious trauma *”) OR (“secondary trauma *”) OR (“compassion fatigue”) OR (“burnout”) OR (“emotional distress”) OR (“resilience”) OR (“resilient”) OR (“psychological resilience”) OR (“emotional resilience”) OR (“compassion satisfaction”) OR (“posttraumatic growth”) OR (“personal growth”)
D—Design	(“questionnaire *”) OR (“survey *”) OR (“interview *”) OR (“focus group *”) OR (“case stud *”) OR (“observ *”) OR (“file review”)
E—Evaluation	(“theme *”) OR (“ thematic analysis”) OR (“self-reflect *”) OR (self-reflex *”) OR (“autoethnographic analysis”) OR (“discourse analysis”)
R—Research type	(“qualitative”) OR (“mixed-method”) OR (“ethnograph *”) OR (“phenomenolog *”) OR (“grounded theory”) OR (“autoethnograph *”)

**Table 2:** Description of included studies (*n* = 30).

Reference (Researcher Discipline)	Researchers: Number % Female (Experience)	Researchers’ Location		Types of Child Abuse Studied	Informant/ Data Source	Sampling Strategy	Design	Data Reduction Strategies
		Country (Number of Researchers/Country)	(Income) Level					
Alexander et al. (1989) [19] (Health Sciences)	*n* = 5 100.0% female (experienced)	USA	High	CSA	File/archive review	Convenience	Cross-sectional	Thematic analysis
Etherington (1996) [57] (Social Sciences)	*n* = 1 100.0% female (experienced)	UK	High	CSA	Adult survivors	Convenience	Cross-sectional	Critical self-reflection
Kinard (1996) [52] (Social Sciences)	*n* = 1 Not specified (experienced)	USA	High	Child abuse (NS)	Children	Convenience	Cross-sectional	Thematic analysis
Skinner (1998) [56] (Organizational Sciences)	*n* = 1 100.0% female (inexperienced)	UK	High	CSA	Caregivers’ reports	Convenience	Cross-sectional	Thematic analysis
Stoler (2002) [60] (Social Sciences)	*n* = 1 100.0% female (inexperienced)	USA	High	CSA	Adult survivors	Convenience	Cross-sectional	Critical self-reflection
Connolly and Reilly (2007) [61] (Social Sciences)	*n* = 2 100.0% female (experienced)	Canada	High	Filicide	Neighbours reports	Convenience	Cross-sectional	Autoethnographic analysis
Coles and Mudaly (2010) [58] (Health Sciences)	*n* = 2 100.0% female (experienced)	Australia	High	Child abuse (NS)	Children, adult survivors	Convenience	Cross-sectional	Critical self-reflection
Scerri et al. (2012) [59] (Social Sciences)	*n* = 1 100.0% female (inexperienced)	Malta	High	CPA, WDV	Adult survivors	Convenience	Cross-sectional	Critical self-reflection
Jackson et al. (2013) [62] (Social Sciences)	*n* = 3 100.0% female (experienced)	UK	High	Child abuse (NS)	File/archive review	Convenience	Cross-sectional	Critical self-reflection
Klocker (2015) [46] (Environmental Sciences)	*n* = 6 83.3% female (experienced)	Australia (1) Tanzania (5)	High Middle	CSA, PA Household poverty Child labour	Children, adult survivors	Convenience	Cross-sectional	Critical self-reflection
ilkes et al. (2015) [63] (Health Sciences)	*n* = 12 91.7% female (experienced)	Australia (11) New Zealand (1)	High High	CSA Orphanhood	Adult survivors	Convenience	Cross-sectional	Thematic analysis
Gabriel et al. (2017) [64] (Health and Social Sciences)	*n* = 7 100.0% female (experienced)	UK	High	Child abuse (NS)	Children, adult survivors	Convenience	Cross-sectional	Critical self-reflection
Powell et al., (2017) [65] (Health Sciences and Law/Criminology)	*n* = 21 71.4% female (experienced)	Australia	High	Child abuse (NS)	Children	Convenience	Cross-sectional	Thematic analysis
Shah (2017) [66] (Social Sciences)	*n* = 1 100.0% female (inexperienced)	UK	High	CSA Intergenerational trauma	Adult survivors	Convenience	Cross-sectional	Autoethnographic analysis
Freya (2018) [69] (Health sciences)	*n* = 1 100.0% female (inexperienced)	Australia	High	CSA WDV	Adult survivors	Convenience	Cross-sectional	Autoethnographic analysis
Rothman et al. (2018) [68] (Health Sciences)	*n* = 4 100.0% female (experienced)	USA	High	Commercially sexually exploited children	Children	Convenience	Cross-sectional	Thematic analysis
Nikischer (2019) [67] (Education)	*n* = 1 100.0% female (experienced)	USA	High	CSA CPA	Children	Convenience	Cross-sectional	Autoethnographic analysis
Adonis (2020) [22] (Social Sciences)	*n* = 1 00.0% female (experienced)	South Africa	Middle	Intergenerational trauma Household poverty	Adult survivors	Convenience	Cross-sectional	Autoethnographic analysis
Guerzoni (2020) [70] (Law/Criminology)	*n* = 1 00.0% female (inexperienced)	Australia	High	CSA	File/archive review	Convenience	Cross-sectional	Autoethnographic analysis
Michell (2020) [71] (Social Sciences and Law/Criminology)	*n* = 1 100.0% female (experienced)	Australia	High	CSA CPA CEA	File/archive review	Convenience	Cross-sectional	Critical self-reflection
Moran and Asquith (2020) [49] (Social Sciences)	*n* = 2 100.0% female (inexperienced)	Australia	High	CSA	Adult survivors	Convenience	Cross-sectional	Critical self-reflection
Williamson et al. (2020) [72] (Social Sciences)	*n* = 10 90.0% female (experienced)	UK	High	CSA	File/archive review	Convenience	Cross-sectional	Autoethnographic analysis
Cullen et al. (2021) [73] (Health Sciences)	*n* = 4 75.0% female (experienced)	Australia (2) Canada (1) UK(1)	High High High	Filicide	Adult survivors, and file/archive review	Convenience	Cross-sectional	Thematic analysis
Sultanić (2021) [74] (Environmental Sciences)	*n* = 21 Not specified (experienced)	USA	High	Unaccompanied child migrants	Children	Convenience	Cross-sectional	Thematic analysis
Gleeson (2022) [75] (Health Sciences)	*n* = 1 100.0% male (experienced)	Australia	High	CSA	File/archive review	Convenience	Cross-sectional	Autoethnographic analysis
Qhogwana (2022) [50] (Social Sciences)	*n* = 1 100.0% female (inexperienced)	South Africa	Middle	CEA CN Filicide	Adult survivors	Convenience	Cross-sectional	Critical self-reflection
Silverio et al. (2022) [51] (Health Sciences)	*n* = 12 91.7% female (experienced)	UK (10) Italy (1) Germany (1)	High High High	CSA Filicide	Caregiver reports	Convenience	Cross-sectional	Critical self-reflection
Alyce et al. (2023) [76] (Health Sciences)	*n* = 1 100.0% female (inexperienced)	UK	High	CSA	Adult survivors	Convenience	Cross-sectional	Critical self-reflection
Reed et al. (2023) [11] (Health Sciences)	*n* = 10 80.0% female (experienced)	USA	High	CSA	Adult survivors	Convenience	Cross-sectional	Thematic analysis
Regehr et al. (2023) [77] (Social Sciences)	*n* = 21 71.4% female (experienced)	Canada	High	Child abuse (NS)	File/archive review	Convenience	Cross-sectional	Thematic analysis

Note: UK = United Kingdom, USA = United States of America, CSA = child sexual abuse, CPA = child physical abuse, Child abuse (NS) = Child abuse not specified, CEA = Child emotional abuse, CN = child neglect, WDV = witnessing domestic violence.

**Table 3:** Synthesis of study findings.

					*n* (%)		*n* (%)
**Risk Factors for Research Induced-Distress**						**Salutary Factors/Outcomes**	
**The researcher domain** **(researcher: characteristics, experience, and biography)**							
***Risk factors for experiencing research induced-distress***						***Salutary researcher characteristics/traits***	
Researcher history of exposure to traumatic life events					18 (60.0)	Experienced researchers (≥5 years research experience)	17 (56.7)
Early career researchers (<5 years research experience)					9 (30.0)	Research commitment	6 (20.0)
						Personal resilience (not specified)	3 (10.0)
						Spiritual beliefs	1 (3.3)
						Flexibility/adaptability	1 (3.3)
**The proximal research domain** **(The nature of the research topic, informal support from team members, and potential threats to researchers’ wellbeing**							
***Lack of social support***						***Social support***	
Researcher working alone					15 (50.0)	Regular team member discussions	19 (63.3)
Lack of support from informal networks					2 (6.7)	Informal support: acquaintances, friends, family	7 (23.3)
No regular team member discussions					1 (3.3)	Researcher working in teams/pairs	1 (3.3)
***Types of child maltreatment***						***Mitigating the impact of exposure to child abuse research***	
Child sexual abuse					20 (66.7)	Informal peer debriefing/discussions	14 (46.7)
Child abuse (not specified)					6 (20.0)	Keeping a research journal	10 (33.3)
Physical abuse					6 (20.0)	Critical self-reflection	10 (33.3)
Filicide					4 (13.3)	Professional counselling	9 (30.0)
Emotional abuse					3 (10.0)	Self-care strategies	7 (23.3)
Child neglect					2 (6.7)	Formal debriefing	7 (23.3)
Witnessing domestic violence					2 (6.7)	Limiting contact time/taking breaks	3 (10.0)
Child labour					1 (3.3)		
Orphanhood					1 (3.3)		
***Dose–response effects***						***Mitigating dose–response effects***	
High daily workloads and/or no regular breaks					9 (30)	Limiting contact time	10 (33.3)
Multiple researcher roles (interviewing/transcribing/coding)					2 (6.7)	Taking regular breaks	10 (33.3)
***Direct contact with abused children or affected adults***						***No direct contact with children or affected adults***	
Interviewing adult survivors of child maltreatment					10 (33.3)	File/archive reviews	7 (23.3)
Interviewing children					7 (23.3)		
Autoethnographies by researchers with a history of child abuse					4 (13.3)		
Interviewing adults about their own child’s maltreatment					3 (10.0)		
***Proximity encounters***						***Accommodating for proximity encounters***	
Researching life-events that the researcher has experienced					15 (50.0)	Critical self-reflection	9 (30.0)
Researching the researchers’ own social minority group					3 (10.0)	Peer discussion/debriefing	3 (10.0)
						Professional counselling	1 (3.3)
***Blurring of boundaries***						***Strategies for maintaining researcher boundaries***	
Researcher and participant roles					11 (36.7)	Critical-self reflection	6 (20.0)
						Peer debriefing	1 (3.3)
						Professional counselling	1 (3.3)
***Threats to researchers’ physical wellbeing***						***Mitigating threats to physical safety***	
Threats to physical safety (not specified)					6 (20.0)	Having and following safety protocols	5 (16.7)
Threat of physical/sexual abuse in the field					2 (6.7)	Conducting research at safe sites	1 (3.3)
Conducting research at unsafe research sites					1 (3.3)		
Researchers’ fears that their children could be abused					1 (3.3)		
***Threats to researchers’ psychological wellbeing***						***Strategies to mitigate threats to psychological wellbeing***	
Distressing emotional states						Mitigating research-induced emotional states	
Sadness/tearfulness					12 (40.0)	Self-copings strategies	
Anger					9 (30.0)	Reducing work load/taking regular breaks	5 (16.7)
Emotional distress (not specified)					8 (26.7)	Engaging in pleasurable activities away from work	5 (16.7)
Feeling anxious					7 (23.3)	Mindfulness, yoga, relaxation exercises	5 (16.7)
Powerlessness					6 (20.0)	Maintaining clear work-home boundaries	2 (6.7)
Emotional exhaustion					6 (20.0)	Compassion satisfaction	
Frustration					6 (20.0)	“Participants benefited from their participation”	6 (20.0)
						“Research findings are meaningful/important”	5 (16.7)
						“The research benefited the researcher”	2 (6.7)
						Posttraumatic growth	
						Personal growth	3 (10.0)
						Acquired resilience	1 (3.3)
Symptoms of traumatic stress						Mitigating symptoms of traumatic stress	
Intrusion symptoms						Informal peer debriefing/discussions	14 (46.7)
Re-experiencing memories of traumatic events					9 (30.0)	Keeping a research journal	10 (33.3)
Distressing trauma-related dreams					7 (23.3)	Critical self-reflection	10 (33.3)
Trauma-related somatic symptoms					5 (16.7)	Professional counselling	9 (30.0)
Avoidance symptoms						Self-care strategies	7 (23.3)
Detachment from trauma-related emotions					5 (16.7)	Formal debriefing	7 (23.3)
Negative alterations in mood and cognitions						Limiting contact time/taking breaks	3 (10.0)
Persistent negative beliefs							
“The world is an unjust/dangerous place”					8 (26.7)		
“I am bad/worthless”					2 (6.7)		
“I have difficulties trusting/being close to others”					1 (3.3)		
Cognitions leading to researcher self-blame							
Guilt (harming/not benefitting participants)					7 (23.3)		
Survivor guilt					4 (13.3)		
Alterations in arousal and reactivity							
Sleep disturbances					12 (40.0)		
Hypervigilance					2 (6.7)		
**The intermediate research domain** **(institutional duty of care, supervision, ethics review committees)**							
***A non-supportive culture of institutional care***						***A supportive culture of institutional care***	
No (or only cursory) initial training/preparation provided					18 (60.0)	Regular ongoing training/debriefing by supervisor during fieldwork	10 (33.3)
A non-supportive/uninformed/uncaring institutional climate					14 (46.7)	Adequate clinical supervision	10 (33.3)
Review boards not addressing potential researcher distress					11 (36.7)	Adequate academic supervision	6 (20.0)
Inadequate clinical supervision					10 (33.3)	Adequate initial training/preparation	5 (16.7)
Researcher primarily responsible for addressing their own distress					9 (30.0)	A supportive/informed/caring institutional climate	4 (13.3)
Supervisor’s ability impaired due to vicarious trauma					1 (3.3)	The academe takes responsibility for addressing researcher distress	3 (10.0)
						Review boards adequately address researcher distress	2 (6.7)
**The distal research domain** **(events/experiences emanating from the broader social system in which academia are embedded)**							
***Consequences of noncompliance with positivist assumptions***						***Support for interpretivist researchers***	
Stoic professionalism (reflecting fear of academe disapproval)					4 (13.3)	Support from colleagues	1 (3.3)
Academe derision of interpretivist researchers					1 (3.3)	Team member discussions	1 (3.3)
Perceived threats to a researcher’s career prospects					1 (3.3)	Adequate training and preparation	1 (3.3)
***Researching macrosystemic/socially mediated trauma***						***Mitigating the impact of macrosystemic trauma***	
Intergenerational/continuing historical trauma involving children					2 (6.7)	Adequate pre-research preparation	1 (3.3)
Household poverty					2 (6.7)	Spacing interviews/taking breaks	1 (3.3)
Commercially sexually exploited children					1 (3.3)	Formal debriefing	1 (3.3)
Unaccompanied child migrants					1 (3.3)	Self-care activities	1 (3.3)
***Cross-cultural challenges***						***Addressing cross-cultural challenges***	
The researcher as a cultural outsider					1 (3.3)	Having cultural brokers/researchers in the research team	1 (3.3)
	Analytic themes		Descriptive themes	Codes (no highlight)			

## Data Availability

Study data are available from the corresponding author on reasonable request.

## Supplementary Materials

The following supporting information can be downloaded at https://www.mdpi.com/article/10.3390/ijerph22030329/s1, Supplementary data S1: Search terms used in database searches; Supplementary data S2: Key journals searched for additional reports; Supplementary data S3: References for studies included in the review; Supplementary data S4: Quality of studies evaluated using JBI-QARI criteria; Supplementary data S5 Verbatim comments by researchers regarding triggers for RID； Supplementary data S6: Verbatim comments by researchers regarding salutary influence on RID outcome.
