# Supplementary material for: Research-Induced Distress Among Qualitative Researchers Who Engage in Research on Child Maltreatment: A Qualitative Systematic Review of Risk and Resilience
Source: Int J Environ Res Public Health. 2025 Feb 23;22(3):329. doi: 10.3390/ijerph22030329 (PMC11942326; doi:10.3390/ijerph22030329)
Supplement: Supplementary file 1 [file ijerph-22-00329-s001.zip › ijerph-3424836-supplementary.pdf]

Research induced distress among qualitative researchers who engage in research on child maltreatment: A qualitative systematic review of risk and resilience

Authors:

Sachet R. Valjee, Steven J. Collings, Denise Rowlett

Supplementary data

Table of contents

#	Description	Page
Introduction	Table of contents	1
(S1)	Search terms used in database searches	2-3
(S2)	Key journals searched for additional reports	4
(S3)	References for studies included in the review	5-7
(S4)	Quality of studies evaluated using JBI-QARI criteria	8
(S5)	Researchers verbatim comments on risk factors for RID	9-14
(S6)	Researchers verbatim comments on salutary influences	15-19

Supplementary data S1: Search terms used in database searches

Scopus

Construct	Search terms	Number of records
#1 Qualitative methods	(“qualitative”) OR (“mixed-method”) OR (“thematic”) OR ("theme*”) OR (“ethnograph*”) OR (“phenomenolog*”) OR (“self-reflect*”) OR (“self-reflex*”) OR (“grounded theory”) OR (“autoethnograph*”)	1,054,096
#2` Researcher	("researcher") OR ("interview*") OR ("transcri*") OR ("coding") OR ("coder*") OR ("interpret*") OR ("translat*") OR ("supervisor") OR ("research team")	214,370
#3 Child maltreatment	("adverse child* experience*") OR ("child* maltreatment") OR ("child* abuse") OR ("child* neglect") OR ("physical neglect") OR ("emotional neglect") OR ("child* physical abuse") OR ("child* sexual abuse") OR ("adult survivors") OR ("molest*") OR ("child* emotional abuse") OR ("domestic violence") OR ("harsh discipline") OR ("corporal punishment") OR (“intergenerational trauma”) OR (“child* traffick*) or (“orphan*”) OR (“community violence”) OR (“peer violence”) OR (bully*”) OR (“discrimination”) OR (“poverty”) OR (“child* homicide”)	436,844
#4 Risk/resilience	(“research-induced distress”) OR (“vicarious trauma*”) OR (“secondary trauma*”) OR (“compassion fatigue”) OR (“burnout”) OR (“emotional distress”) OR (“resilience”) OR (“resilient”) OR (“psychological resilience”) OR (“emotional resilience”) OR (“compassion satisfaction”) OR (“posttraumatic growth”) OR (“personal growth”)	194,842
#5	#1 AND #2 AND #3 AND #4	99

PsycINFO

Construct	Search terms	Number of records
#1 Qualitative research	qualitative OR mixed-method OR thematic OR theme* OR ethnographic OR phenomenology OR phenomenological OR self-reflective OR self-reflection OR self-reflexivity OR self-reflexive OR grounded theory	173,847
#2 Researcher	researcher OR interview* OR transcrib* OR coding OR code* OR interpret* OR translat* OR supervisor OR research team	329,713
#3 Child maltreatment	child* maltreatment OR child* abuse OR child* sex* abuse OR adult survivor* OR child* physical abuse OR molest* OR harsh punishment OR domestic violence OR adverse child* experience* OR child* victim* OR intergenerational trauma OR child* traffick* OR orphan* OR community violence OR peer violence OR bully* OR discrimination OR poverty OR child* homicide	206,404
#4 Outcome	research-induced distress OR vicarious trauma* OR secondary trauma OR compassion fatigue OR burnout OR emotion* distress OR resilien* OR psychological resilien* OR emotion* resilien* OR compassion satisfaction OR posttraumatic growth OR personal growth	42,625
#5	#1 AND #2 AND #3 AND #4 (n =126)	126

MEDLINE

Construct	Search term	Number of records
#1<br>Qualitative research	(MH "Qualitative Research+") or mixed-method* or them*analysis or phenomenolog* or ethnograph* or self-refle* or grounded theory or autoethnog*	93,636
#2<br>Researcher	(MH "Research Personnel") or researcher* or interview* or transcri* or coding or code* or translat* or supervisor* or research team*	923,016
#3<br>Child maltreatment	(MH "Child Abuse+") or child maltreatment or adverse child* experience* or domestic violence or child sexual abuse or child physical abuse or child emotional abuse or physical neglect or emotional neglect or adult survivors or harsh discipline or corporal punishment or intergenerational trauma or child* traffick or orphan* or orphan or community violence or peer violence or bully* or discrimination or poverty or child* homicide	157,725
#4<br>Risk/resilience	(MH "Compassion Fatigue") or (MH "Burnout, Psychological") or (MH "Occupational Stress") or (MH "Stress, Psychological) or (MH "Resilience, Psychological") or research induced distress or vicarious trauma* or secondary trauma* or compassion satisfaction or posttraumatic growth or personal growth	8,246
#5	#1 AND #2 AND #3 AND #4	71

ProQuest

Construct	Search terms	Number of records
#1<br>Qualitative research	(qualitative) OR (mixed-method) OR (thematic) OR (theme*) OR (ethnographic) OR (phenomenology) OR (phenomenological) OR (self-reflective) OR (self-reflection) OR (self-reflexivity) OR (self-reflexive) OR (grounded theory)	292,337
#2<br>Researcher	(researcher) OR (interview*) OR (transcri*) OR (coding) OR (code*) OR (interpret*) OR (translat*) OR (supervisor) OR (research team)	1,013,589
#3<br>Child maltreatment	(child* maltreatment) OR (child* abuse) OR (child* sex* abuse) OR (adult survivor*) OR (child* physical abuse) OR (molest*) OR (harsh punishment) OR (domestic violence) OR (adverse child* experience*) OR (child* victim*) OR (“intergenerational trauma”) OR (“child* traffick*) or (“orphan*”) OR (“community violence”) OR (“peer violence”) OR (bully*”) OR (“discrimination”) OR (“poverty”) OR (“child* homicide”)	100,548
#4<br>Risk/<br>Resilience	(research-induced distress) OR (vicarious trauma*) OR (secondary trauma*) OR (compassion fatigue) OR (burnout) OR (emotional distress) OR (resilience) OR (resilient) OR (psychological resilience) OR (emotional resilience) OR (compassion satisfaction) OR (posttraumatic growth) OR (personal growth)	71,646
#5	#1 AND #2 AND #3 AND #4	291

Supplementary data S2:
Key journals searched for additional reports

Journal focus	Journals searched
Child abuse	Child Abuse & Neglect
	Child Abuse Review
	Child Maltreatment
Qualitative research	International Journal of Qualitative Research
	International Journal of Qualitative Methods
	Qualitative Health Research
	Qualitative Inquiry
	Qualitative Research
	Qualitative Research in Psychology
	Qualitative Research Journal
	Qualitative Social Work
	SSM - Qualitative Research in Health
	The Qualitative Report

Supplementary data S3:

References for studies included in the review

11. Reed, R.M.; Morgan, L.M.; Cowan, R.G.; Birtles, C. Lived experiences of human subjects researchers and vicarious trauma. *J. Soc. Behav. Health Sci.* **2023**, *17*, 164–180. https://doi.org/10.5590/JSBHS.2023.17.1.12
19. Alexander, J.G.; de Chesnay, M.; Marshall, E.; Campbell, A.R.; Johnson, S.; Wright, R. Research note: Parallel reactions in rape victims and rape researchers. *Violence Vict.* **1989,** *4*, 57–62. https://doi.org/10.1891/0886-6708.4.1.57
22. Adonis, C.K. Bearing witness to suffering – A reflection on the personal impact of conducting research with children and grandchildren of victims of Apartheid-era gross human rights violations in South Africa*. Soc. Epistemol*. **2020**, *34*, 64-78. https://doi.org/10.1080/02691728.2019.1681557
46. Klocker, N. Participatory action research: The distress of (not) making a difference. Emotion Space Society **2015**, *17*, 37-44. https://doi.org/10.1016/j.emospa.2015.06.006
49. Moran, R.J.; Asquith, N.L. Understanding the vicarious trauma and emotional labour of criminological research. Innovations 2020, 13. https://doi.org/10.1177/2059799120926085
50. Qhogwana, S. Research trauma in incarcerated spaces: Listening to incarcerated women's narratives. Emotion Space Society **2022**, *42*, 100865. https://doi.org/10.1016/j.emospa.2021.100865
51. Silverio, S.A.; Sheen, K.S.; Bramante, A.; Knighting, K.; Koops, T.U.; Montgomery, E.; November, L.; Soulsby, L.K.; Stevenson, J.H.; Watkins, M.; Easter, A.; Sandall, J. Sensitive, challenging, and difficult topics: Experiences and practical considerations for qualitative researchers. Int. J. Qual. Methods **2022**, *21*, 16094069221124739. https://doi.org/10.1177/16094069221124739
52. Kinard E. Conducting research on child maltreatment: Effects on researchers. Violence Vict. **1996**, *11*, 65–69. https://doi.org/10.1891/0886-6708.11.1.65
56. Skinner, J. Research as a counselling activity? A discussion of some uses of counselling within the context of research on sensitive issues. Br. J. Guid. Couns. **1998**, *26*, 533-540. https://doi.org/10.1080/03069889808253862
57. Etherington, K. The counsellor as researcher: Boundary issues and critical dilemmas. *Br. J. Guid. Couns*. **1996,** *24*, 339–346. https://doi.org/10.1080/03069889608253018

58. Coles, J.; Mudaly, N. Staying safe: Strategies for qualitative child abuse researchers. *Child Abuse Rev*. **2010**, *19*, 56–69. https://doi.org/10.1002/car.1080
59. Scerri, C.S.; Abela, A.; Vetere, A. Ethical dilemmas of a clinician/researcher interviewing women who have grown up in a family where there was domestic violence. *Int. J. Qual. Methods* **2012**, *11*, 102-131. https://doi.org/10.1177/160940691201100201
60. Stoler, L.R. Researching childhood sexual abuse: Anticipating effects on the researcher. *FEM PSYCHOL* **2002**, *12*, 269–274. https://doi.org/10.1177/0959353502012002015
61. Connolly, K.; Reilly, R.C. (2007). Emergent issues when researching trauma: A confessional tale. *Qual. Inq.* **2007**, *13*, 522-540. https://doi.org/10.1177/1077800406297678
62. Jackson, S.; Backett-Milburn, K.; Newall, E. Researching distressing topics: Emotional reflexivity and emotional labor in the secondary analysis of children and young people's narratives of abuse. *SAGE Open* **2013**, *3,* 2158244013490705. https://doi.org/10.1177/2158244013490705
63. Wilkes, L.; Cummings, J.; Haigh, C. Transcriptionist saturation: Knowing too much about sensitive health and social data*. J. Adv. Nurs.* **2015**, *71*, 295–303. https://doi.org/10.1111/jan.12510
64. Gabriel, L.; James, H.; Cronin-Davis, J.; Tizro Kolangarani, Z.; Beetham, T.; Hullock, A.; Raynar, A. Reflexive research with mothers and children victims of domestic violence. *Couns. Psychother. Res.* **2017***, 17*, 157-165. https://doi.org/10.1002/capr.12117
65. Powell, M.B.; Manger, B.; Dion, J.; Sharman, S.J. Professionals' perspectives about the challenges of using interpreters in child sexual abuse interviews. *Psychiatry Psychol. Law* **2017**, *24*, 90–101. https://doi.org/10.1080/13218719.2016.1197815
66. Shah, R. Broken mirror: The intertwining of therapist and client stories of childhood sexual abuse (CSA). *EJPC* **2017**, *19*, 343-356. https://doi.org/10.1080/13642537.2017.1386225
67. Nikischer, A. Vicarious trauma inside the academe: Understanding the impact of teaching, researching and writing violence. *High. Educ*. **2019,** *77*, 905–916. https://doi.org/10.1007/s10734-018-0308-4
68. Rothman, E.F.; Farrell, A.; Bright, K.; Paruk, J. Ethical and practical considerations for collecting research-related data from commercially sexually exploited children. *Behav. Med.* **2018**, *44*, 250–258. https://doi.org/10.1080/08964289.2018.1432550

69. Freya, A. Surviving domestic violence in an Indian-Australian household: An autoethnography of resilience. *TQR* **2018**, *23*, 2686-2699. https://nsuworks.nova.edu/tqr/vol23/iss11/6
70. Guerzoni, M.A. Vicarious trauma and emotional labour in researching child sexual abuse and child protection: A postdoctoral reflection. University of Tasmania. *MIO* **2020**, *13*, 205979912092634. https://doi.org/10.1177/2059799120926342
71. Michell, D.E. Recovering from doing research as a survivor-researcher. *Qual. Rep*. **2020**, *25*, 1377-1392. https://doi.org/10.46743/2160-3715/2020.4048
72. Williamson, E.; Gregory, A.; Abrahams, H.; Aghtaie, N.; Walker, S.J; Hester, M. Secondary trauma: Emotional safety in sensitive research. *J. Acad. Ethics* **2020**, *18*, 55–70. https://doi.org/10.1007/s10805-019-09348-y
73. Cullen, P.; Dawson, M.; Price, J.; Rowlands, J. Intersectionality and invisible victims: Reflections on data challenges and vicarious trauma in femicide: Family and intimate partner homicide research. *JOFV* **2021**, *36*, 619–628. https://doi.org/10.1007/s10896-020-00243-4
74. Sultanić, I. Interpreting traumatic narratives of unaccompanied child migrants in the United States: Effects, challenges and strategies. *LANS-TTS* **2021**, *20*, 227–247. https://doi.org/10.52034/lanstts.v20i.601
75. Gleeson, J. Troubling/trouble in the academy: Posttraumatic stress disorder and sexual abuse research. *High. Educ*. **2022**, *84*, 195–209. https://doi.org/10.1007/s10734-021-00764-x
76. Alyce, S.; Taggart, D.; Sweeney, A. Centring the voices of survivors of child sexual abuse in research: an act of hermeneutic justice. *Front. Psychol*. **2023**, *14,* 1178141. https://doi.org/10.3389/fpsyg.2023.1178141
77. Regehr, C.; Duff, W.; Aton, H.; Sato, C. Grief and trauma in the archives. *J. Loss rauma* **2023**, *28*, 327-347. Https://doi.org/10.1080/15325024.2022.2164143

Supplementary data S4:

Quality of studies evaluated using JBI-QARI criteria

	JBI-QARI Item Number									
**Reference (year)**	**1**	**2**	**3**	**4**	**5**	**6**	**7**	**8**	**9**	**10**
[11] Reed et al. (2023)	Y	Y	Y	Y	Y	Y	Y	Y	Y	Y
[19] Alexander et al. 1989	N	Y	Y	Y	Y	N	Y	Y	N	Y
[22] Adonis (2020) POV	Y	Y	Y	Y	Y	Y	Y	Y	Y	Y
[46] Klocker (2015) POV CL	Y	Y	Y	Y	Y	Y	Y	Y	Y	Y
[49] Moran & Asquith (2020)	Y	Y	Y	Y	Y	Y	Y	?	Y	Y
[50] Qhogwana (2022)	Y	Y	Y	Y	Y	Y	Y	Y	N	Y
[51] Silverio et al. (2022)	Y	Y	Y	Y	Y	?	Y	Y	N	Y
[52] Kinard (1996)	?	Y	Y	?	Y	N	Y	Y	N	Y
[56] Skinner (1998)	N	Y	Y	Y	Y	N	Y	Y	N	Y
[57] Etherington (1996)	Y	Y	Y	Y	Y	Y	Y	Y	Y	Y
[58] Coles & Mudaly (2010)	Y	Y	Y	Y	Y	Y	Y	Y	Y	Y
[59] Scerri et al. (2012)	Y	Y	Y	Y	Y	Y	Y	Y	Y	Y
[60] Stoler (2002)	?	Y	Y	Y	Y	Y	Y	Y	Y	Y
[61] Connolly & Reilly (2007)	Y	Y	Y	Y	Y	Y	Y	Y	N	Y
[62] Jackson et al. (2013)	Y	Y	Y	Y	Y	Y	Y	Y	N	Y
[63] Wilkes et al. (2015)	N	?	Y	Y	Y	N	Y	Y	Y	Y
[64] Gabriel et al. (2017)	Y	Y	Y	Y	Y	Y	Y	N	Y	Y
[65] Powell et al. (2017)	?	Y	Y	Y	Y	Y	Y	Y	Y	Y
[66] Shah et al. (2017)	Y	Y	Y	Y	Y	Y	Y	Y	N	Y
[67] Nikischer (2019)	Y	Y	Y	Y	Y	Y	?	Y	Y	Y
[68] Rothman et al. (2018)	?	Y	?	Y	Y	N	Y	N	Y	Y
[69] Freya (2018)	Y	Y	Y	Y	Y	Y	Y	Y	Y	Y
[70] Guerzoni (2020)	Y	Y	Y	Y	Y	Y	?	Y	N	Y
[71] Michell (2020)	Y	Y	Y	Y	Y	Y	Y	Y	Y	Y
[72] Williamson et al. (2020)	Y	Y	Y	Y	Y	Y	Y	Y	Y	Y
[73] Cullen et al. (2021)	Y	Y	Y	Y	Y	Y	?	Y	N	Y
[74] Sultanić (2021)	?	Y	Y	Y	Y	?	Y	Y	Y	Y
[75] Gleeson (2022)	Y	Y	Y	Y	Y	Y	?	Y	Y	Y
[76] Alyce et al. (2023)	Y	Y	Y	Y	Y	Y	Y	Y	Y	Y
[77] Regehr et al. (2023)	Y	Y	Y	Y	Y	Y	Y	Y	Y	Y

Supplementary data S5

Verbatim comments by researchers regarding triggers for RID

**Domain**	**Triggers for RID**	**Verbatim comments by researchers**
Researcher domain	Lack of preparedness and/or tenure	
	• .< 5-years research experience	*"…in this project, the young co-researchers appeared to be at a loss when faced with traumatic stories during interviews*" [46].
		"Despite extensive clinical experience with rape victims, the nurse researchers found their emotional and behavioral responses to the case material encompassed anger, sadness, anxiety, fear for their own safety, insomnia, nightmares, and nausea" [52].
		"Vulnerabilities identified included age [and] *inexperience"* [58]
		"Only at the end of data collection did I realise there is literature where researchers discuss fieldwork that can be psychologically and emotionally wrenching for investigators regardless of how experienced they are in conducting research" [71].
		"The RAs (research assistants) were untrained in data analysis, being post-graduate students who had not before undertaken any research with sensitive subjects, or had experience coding interview data. Nonetheless, they were asked to code these interviews within a short period of time in order to ensure we remained within our deadlines" [75].
	• Fixed-term or contract researchers	*"…the professional, hurdles I faced…led to the vulnerability of fixed-term and casual staff in shouldering the cost of trauma"* [75].

Domain	Triggers for RID	Verbatim comments by researchers
Proximal Research domain	Secondary victimization	
	• Traumatic countertransference	*"Many of the reactions reported by the researchers—sleeping disorders, emotional changes, somatizing, increased cautiousness, and the need for social support—closely parallel reactions experienced by…victims. The researchers showed a strong tendency toward the same responses and linked their reactions to identifying with the victim"* [19].<br><br>*"In reviewing the cases, I became more and more frightened, as I continued to find young mothers who were attacked in their homes. The threat of harm to their children was often the submitting factor. As a mother, I felt anger and sadness that this happened to these women, and fear in the realization that it could happen to me"* [19].<br><br>*"After reading accounts of child maltreatment, staff voiced feelings of anger, sadness, helplessness, and frustration. They found it difficult to leave the work behind after a day of abstracting records. Thoughts of the children and families continued to trouble them outside of work. Some staff experienced sleep disturbances"* [52].<br><br>*"It was only in retrospect that I realised the severity of the degree of stress I experienced during the interview period; intrusive dreams and images left over from the painful stories I heard day after day…At times I almost lost faith in human goodness"* [57].<br><br>*"The emotional content of the interview had swept me off my feet. I struggled to stand (and to drive) in this sea of emotion that washed around me after the interview. I found myself crying on and off for several days after the interview. I was unable to face the transcription, analysis and coding of this particular interview*" [58].

Domain	Triggers for RID	Verbatim comments by researchers
	• Reactivation of past trauma	"*Anna told me the first time a client brought her story of her childhood abuse, Anna 'froze'. She was stunned at the similarity in their stories: her client was molested by a man, also in a confined space in a public area*". [66]
		"*Rebecca's sense of relating to Jasmine, particularly with regard to the lasting emotional and existential impacts of her abuse experiences, made this interview data particularly traumatic to engage with and tested her emotion regulation skills*". [49]
		"*This is really personal, but my mother is a concentration camp survivor and as a child, she had to flee and was caught. And so, for me, I think that this issue of what's going on right now is excruciating for me in a really deeply personal way*". [74]
		"*…shortly after I began collecting data, I started to have panic attacks and found myself unable to continue recruiting subjects. Subsequently, I developed flashbacks and other symptoms that I eventually recognized as memories of being sexually abused as a child*". [60]
Intermediate/ institutional domain	Inadequate institutional duty of care<br>• Inadequate pre-research training	*"Many participants described their lack of training as being blindsided—where the expectations of engaging with data did not always align with their actual experiences"* [11].<br><br>*"Strategies should be in place to minimise the risk of psychological impact, and quality training and supervision supports researchers to feel adequately equipped to carry out research effectively and sympathetically"* [51].<br><br>*"Vulnerabilities identified included…inadequate preparation for possible impact issues"* [58].

Intermediate/ institutional domain	Inadequate institutional duty of care<br>• Inadequate pre-research training	*"…we recommend that all researchers have appropriate training and awareness of vicarious trauma, as recognising the signs is critical to mitigating the impact"* [73].<br><br>*"The findings of this study…emphasize the need for training and education on trauma-informed interpreting involving both adults and children"* [74].

Domain	Triggers for RID	Verbatim comments by researchers
Intermediate/ institutional domain	• Inadequate supervisor/team leader support	*"Nine of 10 participants wanted support and described the frustration of not getting it, particularly from supervisors. Some of them also discussed how they eventually found support beyond their supervisors"* [11].<br><br>*"I felt misunderstood and alone in my work. The lack of support during the research phase of my work exacerbated my feelings of agitation, anxiety and fear (both that I would experience personal physical harm and that I would emotionally harm my participants…There was no peer or supervisor to talk with. I was completely alone"* [67].<br><br>*"Although senior leaders within the research project did offer to debrief with me, I felt uncomfortable in discussing my experiences of trauma and the impact of the work with them. In large part, this was due to their lack of knowledge in being able to appropriately and efficiently support me—as my diagnosis of PTSD had previously been referred to as my difficulties with the data"* [75].
	• Lack of support from ethics committees	*"Safety of participants was the Ethics Committees' primary consideration. However, while the researchers' physical safety and supervision requirements were addressed, emotional' safety considerations were not directly dealt with"* [58]

Domain	Triggers for RID	Verbatim comments by researchers
Distal research domain	• Stoic professionalism	*"Academics are expected to have control of their emotions and to be professional and rational…I was reluctant to talk to someone about what I had been reading…I was worried I would be criticised, either in a judgmental way for selecting such a topic to study, or for being a complainer or a 'weak' person if I shared my concerns of how the content troubled me*" [70].<br><br>*"The power dynamics at work mean that researchers who are reliant on variable and uncertain work may be reticent to report their experiences of trauma to their manager, for fear of being taken off the research project and losing income"* [75].
	• On being a cultural outsider	*"As a white, middle-class, western researcher I could not know what Tanzanian girls needed"* [46].<br><br>*"A further member had researched sexual and intimate partner violence in diverse cultural settings. For the research assistants involved in the research conversations, transcription and analytical phases elicited emotional responses to the work"* [64].<br><br>*"A decline in interpreter performance…can be heightened in situations where there are cultural taboos preventing transfer of information (e.g., taboos around sexual detail being discussed with the opposite gender)"* [65].
	• On being a cultural insider	*"A subtheme that emerged was how participants were affected when they closely identified with the participant due to sharing similar characteristics, cultures, or experiences. For example, Participant 4 stated "I think that…we have to be more careful when our participants look like us"…"It's so much easier to personalize when the person looks like me or sounds like me."* [11].

Supplementary data S6

Verbatim comments by researchers regarding salutary influence on RID outcome

Domain	Salutary influences	Verbatim comments by researchers
Researcher domain	Salutary characteristics and experiences	
	• Active coping styles	*"After I started to feel a little off, I just made myself stop and take a break. And I really intentionally started doing things like working in my garden and reading happy books and thinking of things in a more positive way. So, I've been really careful about how many qualitative participants I interview at a time. I've been doing psychotherapy and it helps"* [11].
		"The participants developed their own strategies to deal with the effects of transcribing sensitive materials such as online support groups, relaxation activities and unofficial debriefing with friends and family" [63].
		"I have learnt to take my emotional safety seriously. I access clinical supervision; I draw on the support of my colleagues; and also, my family and friends where needed… That anchors me" [73].
	• Resilient traits or resources	*"As researchers we had few risk factors for secondary trauma. We were older, experienced in the clinical fields related to our research, supported and well prepared"* [58].
		"Throughout this autoethnographical piece and my life, resilience has been a recurring theme displayed by my mother, myself and my family" [69].
		"When I was at my lowest point, I began to listen to online spirituality talks as I went to sleep and whenever I woke in the middle of the night. I expected this practice to regenerate energy, which it did" [71].

Domain	Salutary influences	Verbatim comments by researchers
Researcher domain	Salutary characteristics and experiences	
	• Active coping styles	*"After I started to feel a little off, I just made myself stop and take a break. And I really intentionally started doing things like working in my garden and reading happy books and thinking of things in a more positive way. So, I've been really careful about how many qualitative participants I interview at a time. I've been doing psychotherapy and it helps"* [11]. <br>*"The participants developed their own strategies to deal with the effects of transcribing sensitive materials such as online support groups, relaxation activities and unofficial debriefing with friends and family"* [63]. <br>*"I have learnt to take my emotional safety seriously. I access clinical supervision; I draw on the support of my colleagues; and also, my family and friends where needed… That anchors me"* [73].
	• Resilient traits or resources	*"As researchers we had few risk factors for secondary trauma. We were older, experienced in the clinical fields related to our research, supported and well prepared"* [58]. <br>*"Throughout this autoethnographical piece and my life, resilience has been a recurring theme displayed by my mother, myself and my family"* [69]. <br>*"When I was at my lowest point, I began to listen to online spirituality talks as I went to sleep and whenever I woke in the middle of the night. I expected this practice to regenerate energy, which it did"* [71].

Domain	Salutary influences	Verbatim comments by researchers
Proximal research domain	Research-induced resilience	
	• Resilience acquired through research engagement	"*I learned from writing these experiences down and making meaning from my lived understandings of my previous and present self"* [69].
		"I can now appreciate and be thankful for the resilience which I have developed through the PhD, and the ability it provides me to empathise with others who are researching emotionally difficult and sensitive subject areas" [70].
		"Through their search for meaning workers find ways to cope and these become resources for themselves…What worked particularly well for this group was formally provided and appropriate supervision, support and training" [71].
		"When we considered our own goals and the personal impact of doing this work, there was a sense of vicarious resilience that comes with knowing why we do this work and the grounding that comes from working toward enacting systemic and social change" [73].
	• Resilience inspired by participant resilience	*"Carina's interview was…another encounter that proved meaningful for her and for me as well…To me, Carina's way of living her life was a metaphor for resilience, broadly defined as "the capacity to rebound from adversity strengthened and more"* [59].
		"Engaging with George, bearing witness to his story and experiencing his resilience, positively influenced them to start talking about their own past" [64].

Domain	Salutary influences	Verbatim comments by researchers
Intermediate/ institutional domain	• an adequate institutional duty of care	*"With the assistance of the University Ethics Committees', we carefully planned responses to participant distress"* [58].<br><br>*"…supervision was essential to help me process the interviews and help me deal with the intensity of emotions. Supervision was given by the lead supervisor and the co-supervisor, both clinical psychologists and family therapists with extensive clinical experience in the field"* [59].<br><br>*"As team leaders we created…a protocol for data collection, training, and supporting research assistants to collect data over a period of two years, and continually updating procedures and methods as unforeseen challenges were uncovered"* [68].<br><br>*"What worked particularly well for this group was formally provided and appropriate supervision, support and training"* [71].

Domain	Salutary influences	Verbatim comments by researchers
Distal research domain	Mitigating distal influences<br>• Coping despite a perceived need for stoic professionalism	*I serendipitously ran into a former colleague, who offered to be a listening ear if ever I needed it. I took up the offer and we met for coffee one afternoon several weeks later. I was permitted to share of my experience, sorrows, frustrations (at institutions) and anxieties. There was no judgement, only compassion and understanding. This was the starting point for me to process and reconcile all that I had learned, read, felt and heard"* [70].<br><br>*"For us, having a supportive team made a big difference. We could afford for someone to take a couple of days away from fieldwork, because others could pick up that work until the individual had had time to deal with the impacts and return to fieldwork. Where required, we could shift the types of interviews people were conducting. This was only possible because the team worked together in a reciprocal way to support each other"* [72].
	• Including cultural brokers in the study team	*"I refined the research project based on advice from Tanzanian children's rights experts…(and extended)…the 'right' to participate in research projects to non-academics by insisting that affected communities and individuals [including maltreated children] be involved in all possible stages of the research process and associated outcomes"* [46].
